# Clinical, microbiological, and immunological evaluation of patients in corrective orthodontic treatment

**DOI:** 10.1186/s40510-020-00307-7

**Published:** 2020-02-17

**Authors:** Mariana Umekita Shirozaki, Raquel Assed Bezerra da Silva, Fábio Lourenço Romano, Léa Assed Bezerra da Silva, Andiara De Rossi, Marília Pacífico Lucisano, Michel Reis Messora, Magda Feres, Arthur Belém Novaes Júnior

**Affiliations:** 1 Department of Pediatric Dentistry, School of Dentistry of Ribeirão Preto, University of São Paulo, Avenida do Café S/N, Ribeirão Preto, SP 14040-904 Brazil; 2 Department of Oral and Maxillofacial Surgery and Periodontology, School of Dentistry of Ribeirao Preto, University of São Paulo, Avenida do Café S/N, Ribeirão Preto, SP 14040-904 Brazil; 3 Department of Periodontology, Guarulhos University, Guarulhos, SP Brazil

**Keywords:** Gingivitis, Orthodontics, Periodontology, Biofilm

## Abstract

**Background:**

The objective was to analyze clinical, microbiological, and immunological periodontal parameters in patients in corrective orthodontic treatment.

**Materials and methods:**

Twenty-eight patients were selected. Plaque index (PI), bleeding on probing (BOP), width of keratinized gingiva, levels of 40 bacterial species, and of 3 cytokines (IL-1β, MMP-8, and TNF-α) in gingival crevicular fluid (GCF) were evaluated at T0, before orthodontic treatment; T1, 6 months; and T2, 12 months post-treatment. Non-parametric, Friedman, Wilcoxon, ANOVA, and Spearman correlation coefficient tests were used for statistical analyses, with the significance level of 5%.

**Results:**

No significant difference was found for the width of keratinized gingiva, but PI presented a significant increase at T1 and T2 (*p* < 0.05) when compared with T0. The percentage of sites with BOP increased significantly from T0 to T1 (*p* < 0.05); however, at T2, the values decreased and did not differ anymore from T0 (*p* > 0.05). In the microbiological analysis, red complex pathogens were in significantly greater proportions in T2 compared with T0 (*p* < 0.05). There was no statistically significant difference in the cytokine levels between the periods but there was a positive correlation between BOP and IL-1β (*r* = 0.49 *p* = .01) and TNF-α (*r* = 0.39 and *p* = .05).

**Conclusion:**

In conclusion, corrective orthodontic treatment caused clinical periodontal alterations regarding biofilm accumulation and gingival bleeding, with alteration of periodontopathogens.

## Introduction

Periodontal disease is an inflammatory condition that affects supporting tissues of the teeth. Biofilm accumulation is the primary factor associated with the development of gingivitis and periodontitis. According to Socransky et al. [1], the red and orange complex bacterial species are associated with periodontal disease. The orange complex species precede the colonization of the red complex pathogens, *Porphyromonas gingivalis*, *Treponema denticola*, and *Tannerella forysthia*, which are considered the main periodontal pathogens.

In addition to microbiological changes, alterations in the immune system could stimulate an inflammatory response in the tissues and, consequently, an increase in inflammatory cytokines such as tumor necrosis factor (TNF-α) and interleukins (IL-1α, IL-1β, and IL-6). These cytokines and other chemical mediators released during the inflammatory response are able to stimulate collagen destruction via matrix metalloproteinases, triggering attachment loss and a rapid progression of disease [2–4].

Among local factors, orthodontic appliances, fixed and removable, may favor biofilm accumulation and adhesion of cariogenic and periodontal pathogens microorganisms, and, consequently, periodontal diseases and carious lesions development [5, 6]. These clinical changes are mostly related to quantitative and qualitative changes of the oral microbiota. Some studies described the microbial changes in the oral cavity and observed a greater accumulation of cariogenic species and periodontal pathogens, such as species from the genera *Prevotella*, *Bacteroides*, *Fusobacteria*, and *Lactobacillus*, as well as, *T. denticola*, *Eikenella corrodens*, *T. forsythia*, and *Aggregatibacter actionmycetemcomitans* [7, 8].

Many studies aimed to determine the effects of orthodontic appliances in periodontal health and the microbiological composition of the dental biofilm. In general, there was a negative change in clinical parameters, such as excessive biofilm accumulation, greater gingival index, bleeding on probing, gingival enlargement, deepening of pockets and, very often, higher levels of inflammation and gingival bleeding, characterizing the onset of periodontal disease [6, 9–11].

Besides clinical and microbiological parameters, immunological changes can occur in the gingival crevicular fluid after orthodontic appliance bonding. Pathological conditions produce cytokines in response to the presence of microorganisms [12, 13]. Van Gastel et al. [14] observed immunological changes by means of cytokine levels in gingival crevicular fluid (GCF) of patients with orthodontic appliances. The authors showed an increase in clinical indexes and biofilm pathogenicity but there was not a statistically significant difference in cytokine levels. On the other hand, Bergamo et al. [15] analyzed the influence of different types of brackets in the levels of five cytokines in GCF and observed an increase of these cytokines. Giannopoulou et al. [16] also showed an increase in the expression of IL-1β and IL-8 in patients with corrective orthodontic appliances.

Due to this close relation between periodontics and orthodontics, it is of extreme importance to analyze all periodontal changes that patients may present during orthodontic treatment. Although previous studies have assessed the association between orthodontic appliances and several periodontal outcomes such as clinical, host-inflammatory, and microbiological changes in biofilm, no studies to date have thoroughly evaluated all these aspects at the same patients. Thus, the aim of this study was to analyze periodontal parameters in patients during corrective orthodontic treatment, through clinical measurements, microbiological evaluation of 40 bacterial species by checkerboard DNA-DNA hybridization technique, and immunological evaluation with cytokine analyses by a multiplexing analyzer. The null hypotheses tested were as follows: (1) that there is no difference in the periodontal status before and during orthodontic treatment, and (2) there is no correlation between clinical indexes and immunological changes.

## Material and methods

The study was approved by the Experimental Ethics Committee, School of Dentistry of Ribeirão Preto, University of São Paulo, Ribeirão Preto, Brazil (CAAE 56279916.0.0000.5419).

Twenty-eight patients (11–44 years old; mean, 14.35 years) were selected. Patients being treated in the Orthodontic Clinic of the School of Dentistry of Ribeirão Preto, University of São Paulo (Brazil) were selected. The inclusion criteria were as follows: complete permanent dentition, except third molars; no prior orthodontic and periodontal treatments; no antibiotic treatment; and other systemic drugs in the 3 months that preceded the beginning of the study. The exclusion criteria were as follows: patients with periodontitis, skeletal deformity, special needs, and long-term administration of anti-inflammatory medication.

### Sample size calculation

The sample was calculated to provide a power of 80% to recognize a significant difference of 20% in the bleeding index (*δ*) between the times analyzed with a 95% confidence interval (*α* = 0.05) and standard deviation (*σ*) of 0.36, considering the percentage of the bleeding index as the primary variable and [Zα (1.96) + Zβ (0.84)] 2 = 7.84. The calculation was based on the following formula: *n* = [(*σ*) 2/(*δ*) 2] (Zα + Zβ) 2. A total of 25 patients were considered appropriated for this study (after performing the sample calculation). However, considering that some patients could be lost to follow up, 28 patients were enrolled in this study. All patients completed the study.

### Experimental design

Clinical exam and anamnesis were done. The teeth selected were upper and lower first molars and upper and lower left central incisors, based on Periodontal Screening and Recording (PSR), which aims to quickly and simply assess the periodontal conditions of individuals, to identify periodontal health or disease, according to Tekavec and Tekavec [17]. Teeth were analyzed in 3 times: T0, before treatment; T1, 6 months after; and T2, 12 months after bracket bonding. The brackets followed the edgewise standard system: 0.022″ × 0.028″ slot of stainless steel (17.0 to 20.0% chromium, 8.0 to 10.5% nickel, molybdenum 0.60% max) with stainless steel wires (0.016″, 0.018″, 0.020″, or 0.019″ × 0.025″) and 4 bands in the first molars (Morelli, Sorocaba, SP, Brazil). All brackets and tubes used were bonded with composite Transbond XT (3 M Unitek, Monrovia, CA, USA).

Time points T0, before treatment; T1, 6 months; and T2, 12 months that were used in this manuscript were chosen because the great majority of studies used this interval too. Then, result comparison was easier. Another reason was to evaluate if periodontal changes observed in 6 months were maintained in 12 months. Some patients have difficulties to clean appliances in the beginning of treatment. However, after some time they improve their buccal hygiene and obviously the periodontal conditions improve as well. Thornberg et al. [18] analyzed 8 periodontal pathogens before, during, and after orthodontic treatment and observed that 6 of them presented higher counts after 6 months of treatment. These authors stated that 12 months later the levels of these species returned to pre-treatment levels.

### Clinical measurements

Plaque index (PI) was assessed [19] in each patient and determined by the percentage of teeth surfaces with plaque deposits. Six sites were analyzed per tooth (mesiobuccal, buccal, distobuccal, distolingual, lingual, and mesiolingual). Bleeding on probing (BOP) [20] was assessed in the same regions and considered positive when it occurred within 20 s after insertion of the probe for PI evaluation. The width of keratinized gingiva was measured as the distance from the free gingival margin to the mucogingival junction in the selected teeth of each quadrant with millimeter probe.

Kappa index was used to evaluate the examiner calibration on clinical periodontal parameter collection in order to calculate the intra-examiner agreement. The Kappa coefficient greater than or equal to 0.85 was used for examiner calibration. Ten patients, each one showing at least 2 pairs of contralateral multi-rooted teeth, were selected to calibrate the examiner. Each patient was evaluated on 2 separate occasions, 48 h apart in order to obtain the intra-examiner reliability through the Kappa index.

### Microbiological evaluation

Subgingival biofilm samples were collected from each selected tooth at T0, T1, and T2 with individual sterile Gracey curettes. The samples were immediately placed in sterile Eppendorf tubes containing 150 μL of buffer solution (10 mM Tris-HCl, 1 mM EDTA, pH 7.6—TE solution). One hundred microliters of NaOH were added for stabilization of bacterial DNA. The eppendorfs were freezed at − 80 °C. Counts of 40 bacterial species (Table 1) were assessed in each sample, using the checkerboard DNA-DNA hybridization technique, according to Socransky et al. [21] and Mestnik et al. [22].

**Table 1:** Bacterial species strains used on checkerboard DNA-DNA hybridization technique

Species	Strain
*Actinomyces naeslundii* I	12104*
*Streptococcus constellatus*	27823*
*Eubacterium nodatum*	33099*
*Porphyromonas gingivalis*	33277*
*A actinomycetencomitans* (*a + b*)	43817* + 29523*
*F.n.* (sp*. vicentii*)	49256*
*Campylobacter rectus*	33238*
*Treponema socranskii*	S1
*Eubacterium saburreum*	33271*
*Parvimonas micra	33270*
*Veillonella parvula*	10790*
*Actinomyces oris*	43146*
*Streptococcus anginosus*	33397*
*Streptococcus sanguinis*	10556*
*Actinomyces gerencseriae*	23860*
*Streptococcus oralis*	35037*
*Capnocytophaga ochracea*	33596*
*Actinomyces israelli*	12102*
*Streptococcus intermedius*	27335*
*Treponema denticola*	B1¶
*Prevotella nigrescens*	33563*
*Actinomyces odontolyticus* I	17929*
*F.n.* (sp. *polymorphum*)	10953*
*Campylobacter showae*	51146*
*Fusobacterium periodonticum*	33693*
*Neisseria mucosa*	19696*
*Fusobacterium nucleatum* (sp. *nucleatum*)	25586*
*Capnocytophaga gingivalis*	33624*
*Streptococcus gordonii*	10558*
*Tannerella forsythia*	43037*
*Selenomonas noxia*	43541*
*Propionybacterium acnes* (I + II)	11827* + 11828*
*Prevotella melaninogenica*	25845*
*Streptococcus mitis*	49456*
*Eikenella corrodens*	23834*
*Gemella morbillorum*	27824*
*Capnocytophaga sputigena*	33612*
*Leptotrichia buccalis*	14201*
*Campylobacter gracilis*	33236*
*Prevotella intermedia*	25611*

### Checkerboard DNA-DNA hybridization

After collection, the samples were immediately placed in separate Eppendorf tubes containing 0.15 ml of TE (10 mM Tris-HCl, 1 mM EDTA, pH 7.6), and 100 μl of 0.5 M NaOH was added to each tube. Subsequently, the samples were boiled for 10 min and neutralized using 0.8 ml of 5 M ammonium acetate. The released DNA was then placed into the extended slots of a Minislot 30 apparatus (Immunetics, Cambridge, MA, USA), concentrated on a 15/15 cm positively charged nylon membrane (Boehringer Mannheim, Indianapolis, IN, USA) and fixed to the membrane by baking it at 1200 °C for 20 min. The membrane was placed in a Miniblotter 45 (Immunetics) with the lanes of DNA at 90° to the lanes of the device. Digoxigenin-labeled whole genomic DNA probes for 40 bacterial species (Table 1) were hybridized in individual lanes of the Miniblotter. After hybridization, the membranes were washed at high stringency and the DNA probes were detected using the antibody to digoxigenin conjugated with alkaline phosphatase and chemiluminescence detection. The last 2 lanes in each run contained standards at concentrations of 10^5^ and 10^6^ cells of each species. Signals were evaluated visually by comparison with the standards for the test species on the same membrane by a calibrated examiner (*k* test = 93%). They were recorded as follows: 0, not detected; 1, < 10^5^ cells; 2, ~ 10^5^ cells; 3, 10^5^–10^6^ cells; 4, ~ 10^6^ cells; and 5, > 10^6^ cells. The sensitivity of this assay was adjusted to allow detection of 10^4^ cells of a given specie by adjusting the concentration of each DNA probe. The mean counts (10^5^ cells) of individual bacterial species were averaged within each subject and then across subjects. The percentage of the total DNA probe counts was determined initially in each site, then per subject, and averaged across subjects in the 2 groups at each time point. The sum of the individual mean proportion was computed for each microbial complex described by Socransky et al. [1].

**Fig. 1:**
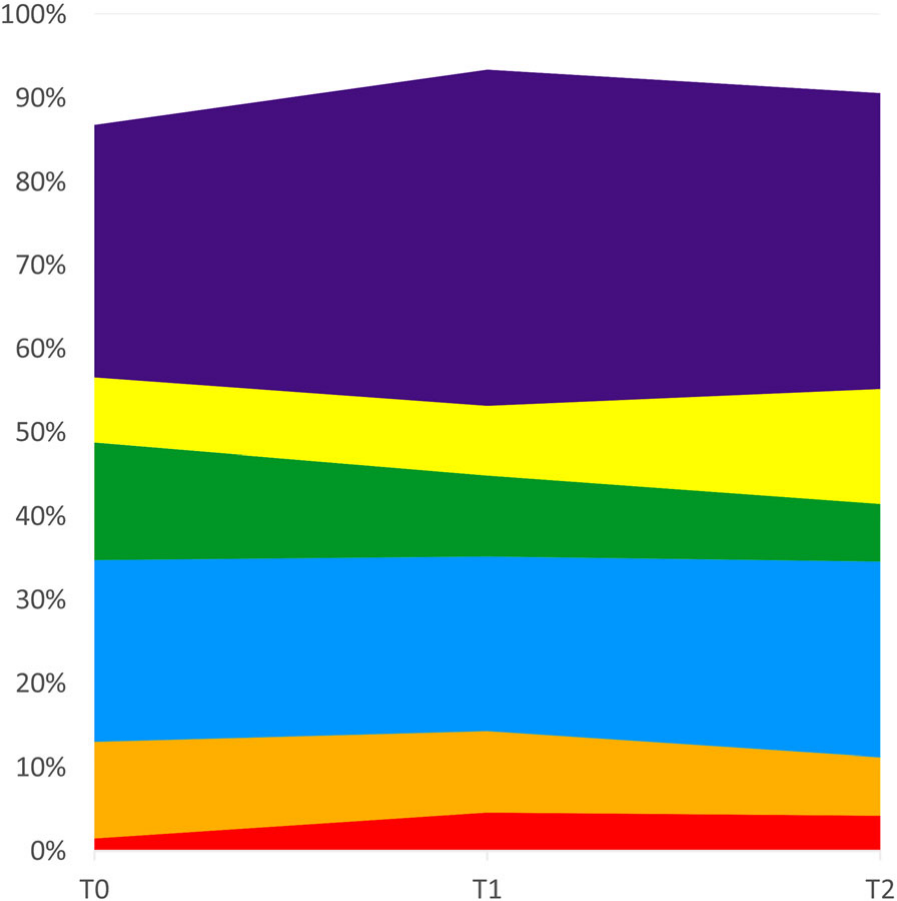
Mean frequencies of each microbial complex at T0, T1, and T2. The colors represent different complexes [1]

### Immunological evaluation

The gingival crevicular fluid was collected using filter paper strips (Periopaper, Oraflow, Inc., Amityville, NY, USA). The strips were placed in the gingival sulcus for 30 s, stored in Eppendorf, and freezed at − 80 °C.

The amount of total protein of each sample was analyzed by conventional enzyme immunoassays (ELISA), with commercially available kits (DC™ Protein Assay, Bio-Rad Laboratories, Inc., Berkeley, CA, USA) following the manufacturer’s instructions. The colorimetric read was done by spectrophotometry in 650 nm (TP-Reader, ThermoPlate®, Brazil) and the values registered in ng/μL.

The quantification of cytokine levels (TNF-α, IL-1β, and MMP-8) was performed with commercially available kits (Milliplex TM Map, Merck Millipore Headquarters, Billerica, MA, USA) and the multiplexing analyzer MAGPIX® (Luminex Corporation, Austin, TX, USA), following the manufacturer’s instructions. All the samples were analyzed individually and the levels of cytokines were estimated from the standard curve using a five-parameter polynomial equation with the software xPONENT® (Luminex Corporation, Austin, TX, USA).

### Statistical analysis

Clinical, microbiological, and immunological parameters were computed for each patient and then averaged. The significance of differences in clinical and microbiological parameters was evaluated using non-parametric methods since the distribution of the variables was not normal. The significance of differences among the three time points was analyzed by Friedman test. When significant difference was achieved, the Wilcoxon test was performed with Bonferroni correction to detect where the difference was. In the statistical analysis of the immunological parameters, the conversion of the data into logarithm was used to compare the cytokine levels between the time points. This was conducted in order to reduce the dispersion and to normalize the variables. After the conversion, ANOVA method was applied for repeated measurements. The Spearman correlation coefficient was used for correlation between clinical indexes and cytokine levels. The level of significance adopted for all analyses was 5%.

## Results

Mean clinical data obtained in the three evaluated periods are presented in Table 2. For PI, mean values were higher at T1 (70.58%) and at T2 (83.23%) when compared with T0 (*p* < .001), but this difference was not statistically significant between T1 and T2. There was a statistically significant increase in the percentage of sites showing BOP from T0 to T1 (*p* = .05). No statistically significant differences were observed in the width of keratinized gingiva between the times (*p* = 0.97).

**Table 2:** Means and standard deviations of clinical parameters at T0, T1, and T2 (baseline, 6 and 12 months) of orthodontic treatment

	Means (%) ± standard deviation			*p* value
	T0	T1	T2	
Plaque index	24.44 ± 11.56^a^	70.58 ± 28.56^b^	83.23 ± 12.30^bc^	*p* < 0.001
Bleeding on probing	4.54 ± 4.98^a^	7.97 ± 5.04^b^	6.20 ± 4.09^ab^	*p* < 0.016
Width of keratinized gingiva	4.29 ± 0.66^a^	4.33 ± 0.84^a^	4.29 ± 0.78^a^	NS

*NS* no significant difference. Different letters indicate a significant difference between the three times (Wilcoxon test)

In the microbiological analyses, over the course of the study, it was observed an increase in *Actinomyces* species (blue group), yellow and purple complexes, and a decrease in the proportions of orange, green, and “other” complexes. However, this variation was not statistically significant (*p* > 0.05). On the other hand, the red complex species (*T. forsythia*, *P. gingivalis*, and *T. denticola*) showed a significant increase in proportions between T0 and T2 (*p* < 0.05). Figure 1 presents the mean proportions of the microbial complexes at the three periods that were analyzed.

**Fig. 2:**
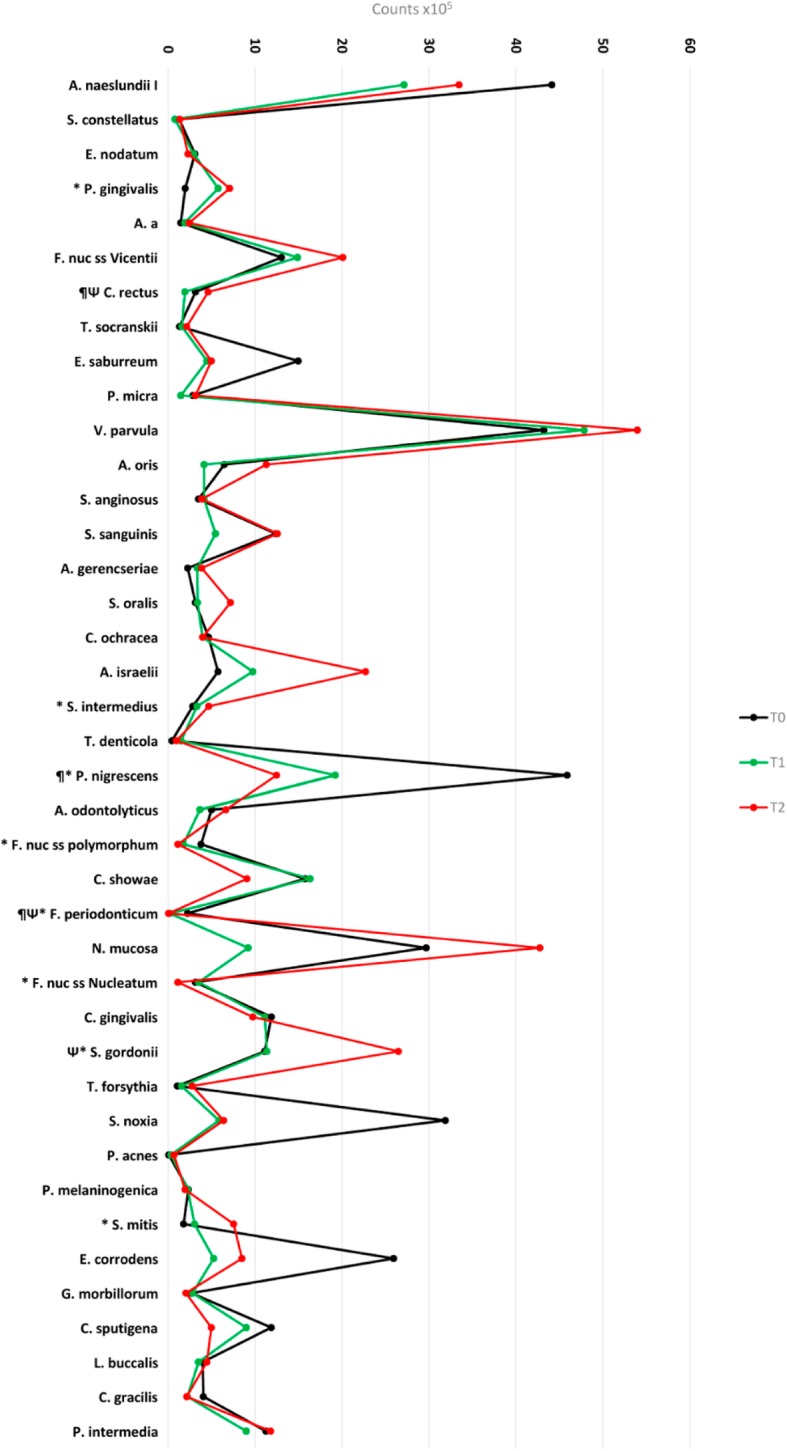
Mean counts (× 10^5^) of 40 bacterial species at T0, T1, and T2. Statistically significant differences: T1 (*) and T2 (¶) when compared with T0; (Ψ) between T1 and T2. Wilcoxon test (*p* < 0,001)

Figure 2 presents the mean total counts (10^5^) of the 40 bacterial species analyzed in the 3 periods (T0, T1, and T2)*.* Nine species changed significantly in levels over the course of the study. Between T0 and T1, there was a decrease in *Campylobacter rectus* (*p* = 0.006), *Prevotella nigrescens* (*p* = 0.05), and *Fusobacterium periodonticum* (*p* = 0.001). Between T0 and T2, there was a statistically significant increase of *P. gingivalis* (*p* = 0.01), *S. intermedius* (*p* = 0.02), *S. gordonii* (*p* = 0.006), and *S. mitis* (*p* = 0.01), and a decrease in *F nuc ss nucleatum*, *F nuc ss polymorphum*, *P nigrescens*, and *F periodonticum* (*p* < 0.001, *p* = 0.001, *p* = 0.02, and *p* = 0.001, respectively). Only *C. rectus* (*p* = .003) and *Streptococcus gordonii* (*p* = 0.05) increased significantly between T1 and T2 (*p* < 0.05).

Table 3 presents the cytokine levels (pg/mL). In general, after the beginning of orthodontic treatment, IL-1β and TNF-α increased at T1 and T2, while MMP-8 decreased; however, all these changes were not statistically significant.

**Table 3:** Cytokine levels (pg/mL) in the gingival crevicular fluid (means ± standard deviation); log expression, at T0, T1, and T2; and baseline, 6 and 12 months

	Means ± standard deviation			*p* value
	T0	T1	T2	
IL-1β	1.1109 ± 0.6558	1.1558 ± 0.6423	1.3411 ± 0.6259	*p* = 0.38, NS
TNF-α	0.3426 ± 0.3392	0.4677 ± 0.3179	0.4707 ± 0.3044	*p* = 0.19, NS
MMP-8	3.8683 ± 1.0388	3.8500 ± 1.0735	3.8481 ± 1.5249	*p* = 0.99, NS

The Spearman correlation coefficient revealed a moderate positive association between BOP and IL-1β (*r* = 0.4, *p* = 0.04) at T0. At T2, BOP presented moderate positive correlations with IL-1β (*r* = 0.49, *p* = −.01) and TNF-α (*r* = 0.39, *p* = 0.05).

## Discussion

The results of the present study showed that patients under orthodontic treatment had a statistically significant increase in PI over a time period of 1 year. These data are in accord to those reported by Abbate et al. [23] who analyzed patients with conventional metallic brackets and observed a significant increase in PI at 6 and 12 months after the beginning of treatment. The greater variations were observed in the first 6 months. In addition, a continuous increase in BOP with a significant difference between 6 and 12 months was observed. In the present study, the percentage of sites with BOP also slightly increased at 6 months, but decreased afterwards. The data for plaque accumulation is very important because biofilm accumulation and poor oral hygiene are associated with poor periodontal conditions, and the orthodontic appliances are considered aggravating factors for periodontal health, which, as a consequence, worsening the periodontal clinical indexes [6, 24, 25].

The microbiological findings showed a progressive increase in the mean proportions of the red complex pathogens and in the levels of *P. gingivalis* from baseline to 12 months. These data are in accordance with Liu et al. [26] who found higher mean counts of *P. gingivalis* in a group of orthodontic patients compared with a control group, without orthodontic treatment. Also, in agreement with our findings, Bergamo et al. [27] observed that patients with self-ligating brackets presented a higher incidence of bacteria of the orange and red complexes 60 days after bracket bonding.

In 2009, Thornberg et al. [18] analyzed 8 periodontal pathogens before, during, and after orthodontic treatment and observed that 6 of them presented higher counts after 6 months of treatment. These authors stated that 12 months later the levels of these species returned to pre-treatment levels. In the present study, among 40 bacterial species, 9 presented statistically significant differences over the course of the study. The majority of the other species evaluated also presented an increase in levels at the 12 months’ time point, but without statistically significance. The exception was *C. rectus* and *F. nucleatum*, which showed a significant decrease at T1 and T2, respectively.

According to several studies, the association between periodontitis and high levels of IL-1β, TNF-α, and MMP-8 in the GCF is well established [13, 28–31]. However, few studies to date analyzed patients with periodontal health and diseases subjected to corrective orthodontic treatment. Gong et al. [10] demonstrated higher levels of IL-1β in a group with gingival enlargement associated with orthodontic treatment in comparison with a control group, periodontally healthy. The authors considered IL-1β a risk factor for the development of gingival enlargement. In the present study, the levels of the cytokines evaluated did not vary significantly between time points.

Giannopoulou et al. [16] showed a statistically significant increase in levels of IL-1β and IL-8 in an orthodontic group, compared with the non-orthodontic treatment group, although no significant changes were observed in PI. There was also a positive association between IL-1β levels and the presence of BOP. In the present study, although the cytokine levels did not vary significantly throughout the study, there was a moderate association between BOP and IL-1β (*r* = 0.4, *p* = 0.04) at the beginning of the study. At 12 months, BOP presented positive correlation with IL-1β (*r* = 0.49, *p* = 0.01) and with TNF-α (*r* = 0.39, *p* = 0.05).

This study showed some findings on cytokine levels in GCF and bacterial species that have not been previously examined. However, it should be considered that assays on GCF are highly variable due to many challenges, and the lack of statistical findings may be a reflection of this variability and inadequate sample size.

In the present study, it was possible to observe that most of the significant changes were in the clinical parameters which can be improved with good oral hygiene instruction during orthodontic treatment. In addition, previous studies concluded that clinical indexes and microbiological parameters in dental crowding are greater than in aligned teeth [32]. Sim et al. [33] observed that the orthodontic treatment group exhibited a lower prevalence of periodontitis compared with the non-orthodontic treatment group, what may due to the fact that tooth alignment that facilitates oral hygiene. However, Agrawal et al. [34] concluded that orthodontic treatment can cause damages to the periodontium, not just in biofilm accumulation and gingival inflammation but also in attachment loss that can occur due to other factors such as tooth extraction and canine movement, tooth movement, incisors inclination, and occlusal trauma during treatment. Also, other factors can interfere with periodontal tissues, such as heavy orthodontic forces and tooth movement which may reduce the alveolar bone thickness and interdental alveolar bone [35, 36].

To the best of our knowledge, this is the first study that evaluated changes in clinical parameters, microbiological profile, and host factors during orthodontic treatment, analyzing changes that occur between time periods, and considering the patient as his own control. However, this study presents some limitations: (1) Absence of data after the end of the orthodontic treatment in order to verify if the periodontal changes occurred up to 1 year would be maintained or would return to normality. (2) Patient/treatment level variables could potentially confound outcomes and regression models for accounting for confounders should be used. (3) This is a single center study, and the findings should be considered generalizable and externally valid with caution.

## Conclusion

The null hypothesis was rejected. Corrective orthodontic treatment caused clinical periodontal alterations regarding biofilm accumulation and gingival bleeding, with alteration of periodontal pathogens.

## Data Availability

The datasets analyzed during the current study are available from the first author (MUS) on reasonable request.

## Abbreviations

BOP: Bleeding on probing
GCF: Gingival crevicular fluid
IL: Interleukin
PI: Plaque index
TNF: Tumor necrosis factor
